# Female reproductive status and ecological conditions impact the magnitude of sex differences in human immune status across the lifespan

**DOI:** 10.21203/rs.3.rs-9510862/v1

**Published:** 2026-05-18

**Authors:** Carmen Hove, Michael D Gurven, Benjamin C Trumble, Jonathan Stieglitz, Daniel Eid Rodriguez, Ivan Maldonado Suarez, Hillard Kaplan, Aaron D Blackwell, Amy M Boddy

**Affiliations:** University of California Santa Barbara; University of California Santa Barbara; Arizona State University; Toulouse School of Economics; Universidad de San Simón; Tsimane Health and Life History Project; Chapman University; Washington State University; University of California Santa Barbara

## Abstract

Current research indicates that women experience lower infectious disease burden and elevated risk of autoimmunity relative to men. Most research, however, is limited to industrialized urban populations and often excludes women in different reproductive phases. We examine age-specific sexual dimorphism in leukocyte differential and neutrophil-to-lymphocyte ratio (NLR), stratified by female reproductive status, among the Tsimane (n = 5,866), a natural-fertility non-industrialized population, and the USA (n = 9,825). We show that sex differences in immune measures across the lifespan are generally lower among the Tsimane (a natural-fertility non-industrialized population) compared to the USA, primarily due to population-specific effects of age and parity on female immune status during pregnancy. Neutrophil-to-lymphocyte ratio, a marker of systemic inflammation, is acutely elevated during pregnancy among primiparous women in the USA, suggesting that later age at first birth and reduced parity may contribute to excess autoimmune disease among women by altering the immunological legacy of pregnancy.

## Introduction

In post-industrial human populations, women experience lower infectious disease burden and lower risk of non-reproductive cancers relative to men, but make up approximately 80% of autoimmune disease diagnoses^1^ and suffer disproportionately from allergy and atopy^2^. These sex biases in disease risk are reflected in sex differences across immune biomarkers^3^. Women generally possess more neutrophils^4^, granulocytes that orchestrate first-line defenses against pathogens, prime antigen-specific immunity, and induce potentially deleterious inflammatory cascades^5,6^. Women also have higher B cell and CD4^+^ T cell counts^7^. Conversely, total lymphocyte count is often higher in males^7^, reflecting higher numbers of regulatory T^8^ and natural killer cells^7^. Consequently, women frequently possess a comparatively higher neutrophil-to-lymphocyte ratio (NLR). NLR is a robust indicator of immune homeostasis and systemic inflammation^9^ strongly associated with the presence and severity of numerous autoimmune disorders that disproportionately affect women (e.g., rheumatoid arthritis)^10–14^. While there is ongoing debate regarding the ultimate selective pressures responsible for sex differences in immune function^15–19^, sex-specific hormone production^20–22^ is an evolutionarily conserved proximate mechanism by which females mount stronger cellular and humoral responses to pathogens^23,24^, vaccination^25,26^, and auto-antigens.

Because female hormone production varies by reproductive state (i.e., premenarchal, regularly menstruating, pregnant, postpartum, and postmenopausal), the direction and magnitude of sex biases in immune function are also expected to vary across these phases. However, the impact of *within-sex* variability on overall sex differences in immune status is not well documented. Likewise, the effect of cumulative reproductive effort (e.g., parity) on female immune function and corresponding sexual dimorphism in immune status is under-explored. The Pregnancy Compensation Hypothesis (PCH) suggests that sex differences in immune function are amplified in low-fertility populations compared to those in the evolutionary past. During pregnancy, the maternal immune system undergoes a compensatory shift, modulated by hormones such as progesterone and estrogens, to support fetal tolerance while maintaining host defense^27,28^. These evolved adaptations are thought to recalibrate the immune system in ways that persist beyond pregnancy. For women with low parity, which is common in industrialized societies, this compensation may become dysregulated, leading to heightened sex differences in immune function and increased susceptibility to immune-related disorders^19^. This hypothesis is supported by evidence that certain autoimmune diseases go into remission during pregnancy^29^ while infectious disease risk is temporarily increased^30^, mimicking a more “male-typical” risk profile. On the other hand, many autoimmune diseases flare or emerge after pregnancy^31^, suggesting that the immunological legacy of gestation is more complicated. To date, however, few studies have investigated the effects of parity on sexual dimorphism in immune function. Furthermore, most research on sex differences in immune function has been conducted in post-industrial or rapidly industrializing populations, where evolutionarily novel conditions, such as reduced microbial exposure and lower energetic demands, may exaggerate underlying sex differences in immunity. These environments limit opportunities for immune system calibration during development and may heighten sensitivity to sex hormones.

In this study, we investigate overall sex differences in immune function as well as variation in immune function among females in different reproductive states across two ecologically distinct populations: a heavily industrialized representative sample from the USA (NHANES) and the Tsimane, a natural-fertility subsistence-oriented population inhabiting the Amazonian river basin^32^. We infer differences in immune activity by examining the distribution of immune cell counts across the lifespan in each population. To test the Pregnancy Compensation Hypothesis^19^, we also estimate and compare population-specific effects of parity on immune markers, stratified by female reproductive state. As indicators of immune status, we focus on white blood cell differentials and neutrophil-to-lymphocyte ratios; these biomarkers reflect broad immunological processes^33,34^ and have well-established diagnostic and prognostic relevance for numerous health outcomes^9,35,36^ (Table 1).

### Female reproduction requires substantial hormonal and immunological shifts

While aging impacts hormone production^37,38^ and immune senescence^39^ in both sexes, female reproduction requires additional hormonal changes and presents unique immunological challenges. During pregnancy, production of estradiol, estrone, testosterone, and progesterone are significantly elevated above baseline^40^. These hormonal shifts support fetal development^41^ and fetal tolerance, a phenomenon in which the maternal immune system must tolerate fetal antigens while maintaining essential immune defenses^42,43^. Following delivery, ovarian function shifts again: estrogen and progesterone production are suppressed by the antagonistic effects of prolactin, a hormone which remains elevated during regular lactation. There is some evidence that pregnancy produces lasting alterations in production of certain hormones, including reduced prolactin secretion^44^ and elevated estriol^45^, but how these long-term alterations impact immune function is not well understood. Finally, menopause marks the end of the female reproductive lifespan, with the ovaries switching to low production of estrogens and progesterone and continued production of androgens^46^.

The dynamic nature of ovarian function over the life course suggests that hormone-mediated sex biases in immune function and disease risk should be most pronounced between puberty and menopause. Given the steep increase in estrogens and progesterone during gestation and evidence that systemic inflammation *increases* during pregnancy^47^, sex bias in immune measures may be further accentuated by pregnancy. In line with these predictions, autoimmune diseases that most disproportionately affect women (e.g., primary Sjögren’s syndrome, systemic lupus erythematosus, Hashimoto thyroiditis) are most commonly diagnosed among women between 20 and 50 years of age^48–50^ and often emerge or worsen after pregnancy^51^. On the other hand, immunological shifts induced by fetal and placental cues during pregnancy may *reduce* sex differences in immune measures^19^. Regular exclusion of pregnant women and failure to consider the effects of parity preclude a clear picture of how age, current female reproductive status, and female reproductive history combine to impact sex differences in immune status.

### Hormone production and immunological development are sensitive to ecological inputs

Socio-ecological conditions that increase energy balance (e.g., caloric excess, sedentary behavior) may magnify hormone-mediated sex differences in immune function via disproportionately large effects on female ovarian function. Because female reproduction across mammalian species requires significantly greater energetic investment than male reproduction, female ovarian function is responsive to energy balance. While long-term reductions in energetic availability suppresses baseline ovarian function, resulting in lower progesterone production across the ovarian cycle^52–54^, positive energy balance and reduced metabolic load are linked to earlier age at menarche^55^, elevated adult levels of estradiol^56^ and progesterone^53^, higher progesterone levels during the peri-ovulatory and peri-implantation period^54^, and shorter duration of lactational amenorrhea among breastfeeding women^57^. While moderate energetic availability is associated with enhanced fecundity^58^ and greater immune competence^59^, caloric excess contributes to chronic inflammation and greater risk of hypersensitivity to a broad range of antigens^60^. Furthermore, exposure to *exogenous* female sex hormones via regular use of oral contraceptives, an evolutionarily novel feature of industrialized societies, has also been linked to immunological hypersensitivity to a variety of antigens - including steroid hormones themselves^61^.

Lack of exposure to certain pathogens during development may also increase sexual dimorphism in immune function, not just by increasing the amount of energy allocated to sex-specific hormone production, but by reducing the number of opportunities for immunological calibration. In the USA, for example, reductions in infectious disease burden^62^ have increased life expectancy^63^ but are linked to the rise of chronic inflammatory disorders characterized by hypersensitivity to non-pathogenic antigens (e.g., atopy, autoimmune disease)^64^. One explanation for this relationship is that exposure to pathogens during development primes the immune system to differentiate between pathogenic and non-pathogen antigens and, in certain cases, induce immunological tolerance. People living in rural, non-industrialized contexts characterized by elevated pathogen load exhibit higher baseline levels of most immune markers^65,66^ but seemingly low incidence of allergy or autoimmune disease^67–70^. Lack of calibrating opportunities via pathogen exposure may have particular relevance during pregnancy, when the maternal immune system must induce tolerance of fetal antigens. Current evidence indicates that non-industrialized populations experiencing high pathogen exposure exhibit less immunological activation (e.g., lower peak in neutrophil count and C-reactive protein) during pregnancy^47^, which may reflect both lower hormone production and reduced immunological sensitivity to fetal antigens during gestation.

Taken together, these patterns suggest that evolutionarily novel environmental conditions common to industrialized populations (e.g., low energetic throughput, microbial deprivation) may exacerbate evolved sex differences in immunity by reducing opportunities for immunological calibration during development and increasing exposure and/or sensitivity to sex hormones - especially among females. To date, however, these ideas remain largely unexplored and therefore current definitions of “normal” sexual dimorphism in immune status rest primarily on studies conducted within industrialized/industrializing societies (e.g., China, the USA, India).

### Objectives and predictions

We utilize data from the Tsimane, a natural-fertility subsistence population inhabiting the Amazonian River basin, and a representative sample from the United States (NHANES), to estimate the age-dependent effects of sex on white blood cell counts and neutrophil-to-lymphocyte ratio using generalized additive models. We stratify by female reproductive phase (premenarchal, regularly cycling, pregnant, 0–12 months postpartum, and postmenopausal). Among postmenarchal females, we also estimate the population-specific effects of parity on immune measures, stratified by female reproductive state.

Within both populations, we predict that (a) sex biases in immune status will be most pronounced for women of reproductive age, with (b) pregnancy in particular corresponding to greater sexual dimorphism relative to other reproductive states. Given the reversal of pregnancy-induced hormonal patterns and suppression of ovulation following delivery, we expect that (c) postpartum females will exhibit less divergent immune profiles from their male counterparts. Likewise, we hypothesize that (d) sex differences in immune status will be attenuated or reversed among postmenopausal females and their male peers, due to the combined effects of aging in both sexes and menopause among females. Regardless of female reproductive state, we expect that (e) sex biases in immune measures will be attenuated among the Tsimane compared to the USA. Finally, we test the Pregnancy Compensation Hypothesis’ core prediction that (f) reduced parity will be associated with a larger degree of sex bias in industrialized populations^19^.

## Results

### Female reproductive status has age-dependent and population-specific effects on the direction and magnitude of sex differences in immune markers

**Figure 1:**
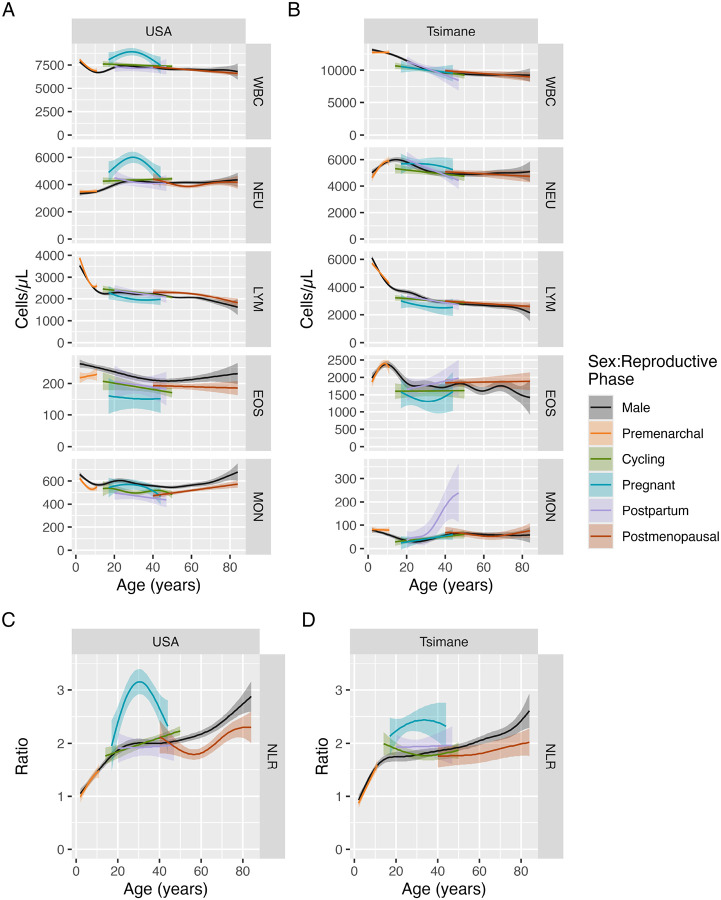
Non-linear effects of age by sex and female reproductive phase on (A) leukocyte differential among the Tsimane, (B) leukocyte differential in the USA, (C) NLR among the Tsimane, and (D) NLR in the USA. Estimated values are standardized by parity (set to 0 live births for premenarchal females and 3 live births for regularly cycling, pregnant, postpartum, and postmenopausal females) and z-scored BMI (set to 0). Solid lines correspond to estimated mean value; shaded regions correspond to 95% credible intervals. Please note that each variable is plotted on its own scale.

Among 8,624 males in both populations (Tsimane n = 3,205; USA n = 5,419), age is associated with strong non-linear and population-specific effects on all immune measures, especially within the first two decades of life (Fig. 1, Supplementary Table 4, Supplementary Table 5). Among 7,067 females (Tsimane n = 2,661; USA n = 4,406), the effects of age on immune measures are mediated by current reproductive state and differ based on population and immune measure (Table 1, Fig. 1, Supplementary Table 4, Supplementary Table 5).

### In both populations, sex biases in certain immune measures emerge before females reach menarche but maximum sex differences in all immune markers are observed during the reproductive years

We find no differences in neutrophil count, total white blood cell count, or NLR between premenarchal females and their male peers in either population, regardless of age (Fig. 1A, Fig. 1C, Table 2). We do, however, find sex differences in total lymphocyte and monocyte counts between premenarchal females and age-matched males at certain ages, but the direction of these biases differs between populations.

In the USA, premenarchal females exhibit slightly *higher* total lymphocyte counts from ages 2 to 4 and again at age 11, with a maximum difference of 10% (95% CI: 7%, 13%) at age 2 (Supplementary Table 3). Premenarchal females in the USA also possess *lower* monocyte counts between the ages of 3 and 9, with a maximum difference of 10% (95% CI: 7%, 12%) occurring at age 7 (Supplementary Table 3). Among the Tsimane, premenarchal females have slightly *lower* total lymphocyte counts, but only from ages 2 to 3, with a maximum difference of 6% (95% CI: 4%, 9%) at age 2. Likewise, premenarchal females possess *higher* monocyte counts than males, but only from ages 10 to 11, with a maximum difference of 37% (95% CI: 15%, 59%) at age 11. In the USA, premenarchal females also exhibit *lower* eosinophil counts than males between the ages of 2 and 9, with a maximum difference of 17% (95% CI: 10%, 24%) in estimated cell counts occurring at age 2 (Supplementary Table 3). No such pattern is found among the Tsimane.

As predicted, maximum sex differences in white blood cell count, neutrophils, eosinophils, and NLR are more pronounced between regularly cycling females and their male counterparts relative to the differences between premenarchal females and males. This pattern holds across both populations. Unexpectedly, however, the direction of these biases varies between populations for certain measures (i.e., neutrophils and total white blood cell counts). Furthermore, the ages at which sex biases occur between regularly cycling women and men are more limited than expected across most immune markers.

As shown in Tables 2 and S3, regularly cycling females in the USA have higher neutrophil and total white blood cell counts than males at both younger and later ages, with a maximum difference of 17% (95% CI: 10%, 25%) and 12% (95% CI: 8%, 17%), both occurring at age 14 (Supplementary Table 3). Likewise, there is a female bias in NLR between regularly cycling females in the USA and their male counterparts, but only from ages 43 to 50, with a maximum difference of 8% (95% CI: 4%, 12%) at age 49. Most ages, however, are characterized by an absence of robust sex biases in these immune measures. Conversely, regularly cycling females in the USA have lower eosinophil counts than males at all ages, with a maximum 16% (95% CI: 4%, 27%) difference observed at age 14. Regularly cycling females also exhibit lower monocyte counts between ages 19 and 50, with a maximum difference of 15% (95% CI: 11%, 19%) at age 26 (Supplementary Table 3, Fig. 1A).

Among younger individuals, regularly cycling Tsimane women have lower neutrophil, total lymphocyte, eosinophil, and total white blood cell counts and higher NLR than males, with maximum differences reaching 12% (95% CI: 6%, 17%), 16% (95% CI: 10%, 22%), 28% (95% CI: 18%, 38%), 13% (95% CI: 9%, 17%), and 18% (95% CI: 5%, 31%), respectively (Supplementary Table 3). Most ages, however, are characterized by an absence of robust sex biases in these immune measures (Fig. 1B, Table 2).

### Female sex bias in NLR across the reproductive lifespan is predominantly driven by pregnancy in both populations - but this effect is greater and more sustained in the USA

As predicted, we find that the largest sex differences in total lymphocyte and NLR across the lifespan occur between pregnant women and men. This pattern is found in both the USA and among the Tsimane.

Pregnant females in the USA have higher neutrophil count, total white blood cell count, and NLR than males for the majority of ages represented, with highly non-linear effects of age producing maximum sex differences of 41% (95% CI: 31%, 50%), 21% (95% CI: 16%, 26%) and 57% (95% CI: 45%, 69%) at 29, 30, and 29 years of age, respectively (Fig. 1A, Table 2, Supplementary Table 3). Conversely, pregnant females in the USA have lower total lymphocyte count than males from ages 31 to 41, with a maximum difference of 13% (95% CI: 5%, 21%) at age 38, and lower eosinophil counts between ages 17 and 41, with a maximum difference of 33% (95% CI: 9%, 57%) at age 17.

Pregnant Tsimane females have lower total lymphocyte count than males from ages 17 to 31, with a maximum difference of 22% (95% CI: 13%, 31%) observed at age 21. This negative effect of pregnancy on total lymphocyte count results in a substantial female bias in NLR between pregnant Tsimane women and men, with a maximum difference of 36% (95% CI: 24%, 48%) occurring at age 31. Pregnant Tsimane women also exhibit lower eosinophil counts than age-matched men between ages 24 and 32 and lower total white blood cell count from ages 17 to 21, with maximum differences of 26% (95% CI: 11%, 42%) and 11% (95% CI: 3%, 19%) occurring at ages 30 and 17, respectively (Supplementary Table 3).

### In both populations, monocytes are the only immune marker that substantially varies between postpartum women and age-matched men

As predicted, we find no significant sex differences in total white blood cell count, neutrophils, total lymphocytes, eosinophils, or NLR among postpartum women and age-matched men in either population. There is, however, an age-dependent male bias in monocyte counts in the USA and a robust, age-dependent female bias among the Tsimane. Postpartum females in the USA have lower monocyte counts than males from ages 20 and 47, with a maximum difference of 19% (95% CI: 11%, 27%) at age 24. Postpartum females in the Tsimane have *higher* monocyte counts than males from ages 35 to 47, with a maximum difference of 266% (95% CI: 73%, 460%) and age 47.

### After menopause, sex differences in immune markers are generally reversed or absent in both populations

Depending on age, postmenopausal females in the USA possess *lower* neutrophil, eosinophil, and monocyte counts and NLR and *higher* total lymphocyte count than males across a substantial portion of the post-reproductive lifespan (Fig. 1A, Table 2). Sex differences in neutrophil, eosinophil, and monocyte cell counts peak at 7% (95% CI: 4%, 10%), 16% (95% CI: 9%, 24%), and 15% (95% CI: 10%, 19%) at ages 58, 75, and 83, respectively (Supplementary Table 3).

Depending on age, postmenopausal Tsimane women have *lower* NLR and eosinophil counts than males, reaching a maximum difference of 23% (95% CI: 13%, 32%) and 16% (95% CI: 8%, 24%) at ages 84 and 59, respectively (Fig. 1, Table 2).

### Higher parity corresponds with reduced sex bias in neutrophil count and NLR, but only among pregnant women in the USA

**Figure 2:**
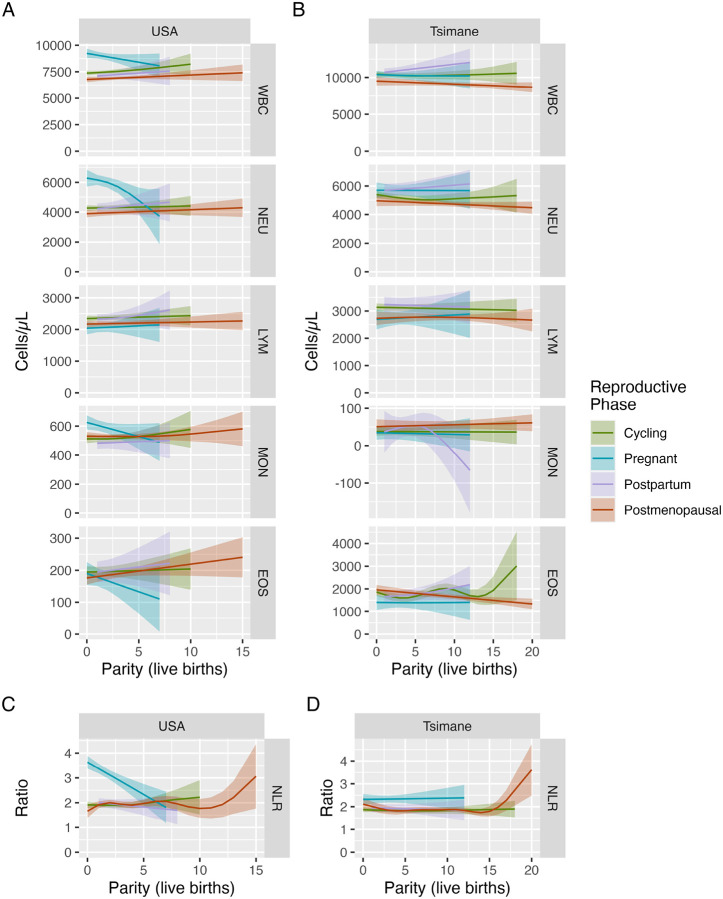
Non-linear effects of parity by female reproductive phase on (A) leukocyte differential among the Tsimane, (B) leukocyte differential in the USA, (C) NLR among the Tsimane, and (D) NLR in the USA (Table 1). Estimated values are standardized by age (set to 24 years for regularly cycling, pregnant, and postpartum females and set to 65 years for postmenopausal females) and z-scored BMI (set to 0). Solid lines correspond to estimated mean value; shaded regions correspond to 95% credible intervals. Please note that each variable is plotted on its own scale.

We do not find any robust effects of parity on immune markers among regularly cycling, pregnant, or postpartum Tsimane females. Likewise, we do not find strong effects of parity on immune measures among regularly cycling or postpartum females in the USA (Fig. 2, Supplementary Table 4, Supplementary Table 5). We do, however, find strong negative effects of parity on neutrophil count (F = 4.266; P-value = 0.009), monocyte count (F = 2.874; P-value = 0.090), and NLR (F = 20.734; P-value = < 0.001) among pregnant females in the USA (Fig. 2, Supplementary Table 5). Consequently, high-parity pregnant women in the USA exhibit immune profiles that are more similar to age-matched men compared to primiparous women. Controlling for BMI and age, the estimated neutrophil count, monocyte count, and NLR for a *currently pregnant* nulliparous woman in the USA is 6,286 cells/μL (95% CI: 5,727–6,845), 626 cells/μL (95% CI: 577–674), and 3.63 (95% CI: 3.38–3.89), respectively. A pregnant woman with four prior live births therefore has a 15% (95% CI: 7%, 24%) lower neutrophil count, a 12% (95% CI: 3%, 22%) lower monocyte count, and 26% (95% CI: 18%, 34%) lower NLR compared to a currently pregnant nulliparous woman of the same age.

Among postmenopausal females in both populations, we find a statistically significant non-linear effect of parity on NLR, wherein there are positive effects of parity on NLR but only at higher parity values (Fig. 2, Supplementary Table 4, Supplementary Table 5). Given the relatively small sample size of women who have > 10 live births, the credible intervals for NLR values at high parity values are wide and should be interpreted with caution. Among postmenopausal Tsimane females, we also observe a slight negative effect of parity on eosinophil count (F = 9.941; P-value = 0.002). Controlling for BMI and age, a postmenopausal Tsimane woman with a history of four live births has a 7% (95% CI: −4%, 18%) lower eosinophil count than an age-matched nulliparous postmenopausal woman.

## Discussion

The results of this study show that age, current female reproductive state, and female reproductive history (e.g., parity) influence the direction and magnitude of sex differences in immune markers across the lifespan. Furthermore, population-level comparisons between the USA and the Tsimane strongly suggest that sexual dimorphism in certain immune markers, especially those related to systemic inflammation and general immune activation, are exaggerated within heavily industrialized societies. More specifically, our findings indicate that population-level differences may be largely (but not entirely) driven by divergent inflammatory responses to pregnancy.

Pregnancy is often described as a period during which autoimmune disease risk is temporarily alleviated while risk of certain infections is higher, resembling a more male-typical risk profile. The results of this study, on the other hand, indicate that pregnancy is a primary driver of sex differences in immune cell counts, especially in industrialized populations. In both the USA and the Tsimane, we find that sexual dimorphism in neutrophil-to-lymphocyte ratio (a simple yet robust indicator of immunological homeostasis) is most pronounced between pregnant women and age-matched men, with pregnant women exhibiting substantially higher NLR. Among pregnant women in the USA, we find robust non-linear effects of age and strong negative effects of parity on neutrophil count and NLR. In the USA, estimated neutrophil count and NLR are therefore highest among primiparous pregnant females around 29 years of age, with NLR values exceeding pre-established thresholds of “mild to moderate inflammation”^71^.

Supporting some of the predictions of the Pregnancy Compensation Hypothesis, we do not observe these same effects of age or parity among pregnant Tsimane women, and therefore maximum sex differences (as well as within-sex variation) in neutrophil count and NLR among the Tsimane are much smaller than those observed in the USA. For example, in the USA we find that predicted NLR among pregnant women can be up to 57% (95% CI: 45%, 69%) higher than the NLR values observed among men, depending on age and parity, while this pattern is relatively attenuated among the Tsimane (up to an estimated ~ 36% female bias). However, the mechanisms driving these sex-differences in the US are more complicated and these results suggest a refinement of the Pregnancy Compensation Hypothesis, including considerations of age at first birth. At a mechanistic level, high NLR among women in the USA who become pregnant for the first time in their late-twenties to early-thirties may be due to elevated baseline estrogen levels and altered progesterone-to-estradiol ratio. Studies among non-pregnant women in industrialized populations report a non-linear effect of age on estradiol production, with levels peaking around the age of 30^72^. The absence of strong age effects on immune status among pregnant Tsimane women may reflect lower age-related variability in hormone production due to chronic non-reproductive demands on energy allocation. The absence of strong parity effects on immune measures among the Tsimane suggest that the impact of cumulative reproductive output on female immune function may depend on the broader ecological context.

According to the 2023–2024 Centers for Disease Control, the average woman in the USA gives birth for the first time at 27.5 years old^73^ and has approximately 1.8 live births over her lifespan^74^. These national trends are remarkably close to the demographic that we find to have acutely elevated NLR during pregnancy. Given the association between high NLR and immunological dysregulation^11,13,75^, it is tempting to speculate that widespread changes in reproductive behavior within industrialized societies (later age at first birth and reduced parity) contribute to excess autoimmune diseases diagnoses among women by altering the immunological legacy of pregnancy. If true, this may explain why many autoimmune diseases flare and/or emerge after pregnancy^31^ and why, when lumped together, women are predominantly diagnosed with these diseases before the average age of menopause^76^. During pregnancy, negative effects of acutely elevated NLR on overall disease risk may be mitigated by the presence of the fetus/placenta and the associated mechanisms that induce fetal tolerance (e.g., regulatory T cell proliferation, regulation of neutrophil phenotypes)^27,77^. Deleterious effects may then emerge after delivery, when placental cues are removed but offspring cells often remain in the maternal body^29^. Relatively low rates of extended on-demand breastfeeding after delivery^78^, an evolutionarily conserved phase of mammalian reproduction often described as the “4th trimester”, may further magnify these effects by impeding postpartum immunological recovery^79^. We recommend that future research investigate the more granular effects of time since delivery and breastfeeding behavior on immune function, as this approach may illuminate important effects that we were not able to separate out in this study.

Another way future studies can evaluate the immunological legacy of pregnancy is by comparing short, mid, and long-term health outcomes among women with different reproductive histories. Given that we find comparatively little sexual dimorphism between regularly cycling females and their age-matched male counterparts and only minor effects of parity on immune status among postmenopausal females, it is possible that nulliparous women without any underlying fertility challenges have a *reduced* chance of developing autoimmune disease compared to low-parity women who begin their reproductive careers in their late-twenties or early-thirties. Likewise, our results suggest that risk for autoimmune disease onset following pregnancy may be lower among women who start their reproductive career relatively early. We strongly recommend that future studies consider both age at first birth and total number of pregnancies when investigating sex differences in health outcomes.

While pregnancy is a primary driver of sex differences in immune status in both populations, our results indicate that the transition to menopause is marked by sexual dimorphism in immune status in the USA but not among the Tsimane. In the USA, we find a sustained male bias in neutrophil, eosinophil, and monocyte count and NLR and a female bias in total lymphocyte count among postmenopausal women and age-matched men. In contrast, we find a near-absence of sex differences in immune measures among postmenopausal Tsimane women and their male counterparts. These patterns suggest that the transition to menopause is characterized by a more severe drop-off in ovarian hormone production among females in the USA and/or greater sensitivity of the immune system to the hormonal changes that occur during menopause. In sum, this study shows that women in the USA (especially those who have one or more pregnancies) experience a much higher degree of overall variability in certain immune measures (e.g., neutrophil count and NLR) across the lifespan when compared to Tsimane women, presumably due to greater vacillations in ovarian sex hormone production. Notably, this within-population variation in immune measures among US women, which is driven by reproductive state, parity, and age, exceeds the magnitude of sex differences observed between women and age-matched men. In other words, reproductive history explains the differences in immune measures more than sex - but only in the US.

Lastly, we find a consistent male bias in eosinophil and monocyte count across nearly all ages and female reproductive states within the USA but observe no such bias among the Tsimane. These results indicate that monocyte and eosinophil production is less responsive to changes in ovarian sex hormone production across lifespan. Furthermore, the absence of a sustained male bias in eosinophil and monocyte count among the Tsimane suggests that sex differences in these immune measures are not universal. These population differences may arise from the comparatively lower infectious disease burden in the USA, which could unmask sex-specific effects of gene expression on immune development.

### Additional Considerations

While useful in establishing a general understanding of immune status, variability in cell counts does not perfectly map on to variation in cellular function or underlying gene expression. Future comparative work is needed to investigate the effects of age, sex, female reproductive status, parity, and ecological conditions on a broader range of immune markers (e.g., oxidative bursts and release of extracellular traps by neutrophils). We especially recommend that future research focus on neutrophil subtypes, especially low-density neutrophils, as these have been identified as key players in the etiology of autoimmune disease^80^.

Additionally, the comparisons we draw between the USA and the Tsimane should be interpreted in context. There is significant variation in socio-ecological conditions *within* non-industrialized societies that must be carefully considered, including differences in physical environment (e.g., altitude, seasonality, type of pathogen exposure), social structure (e.g., population density, matriarchal versus patriarchal customs), and physiology. For example, the Shuar (another tropical South American Indigenous population subsisting on horticulture and foraging) have distinct pathogen exposure profiles compared to the Tsimane^65^. Furthermore, there is considerable socio-ecological variation within industrialized societies (e.g., population density, socio-economic status) that we chose to collapse based on the aims of this particular study. Lastly, we did not include concurrent use of hormonal birth control among regularly cycling women as a co-variate in our models due to a high proportion of missing values in the NHANES dataset. Future studies focusing exclusively on women in the USA who have never used hormonal birth control may be especially useful for teasing out the effects of exogenous hormone exposure common within industrialized populations.

## Conclusions

In this study, we show that pregnancy is a primary driver of sex differences in neutrophil and NLR immune cell counts in two socio-ecologically distinct populations. As predicted by the Pregnancy Compensation Hypothesis, we find these sex differences in immune cell counts are more pronounced within the USA when compared to the Tsimane. Considering the impact of age on immune cell counts of pregnant females in the USA, we suggest further refinement of the hypothesis to consider the timing and tempo of reproductive history in females. Given the link between acutely elevated NLR and heightened risk of autoimmunity, we propose that later ages at first birth and lower parity may contribute to excess autoimmune diseases diagnoses among women in high-income countries by altering the immunological legacy of pregnancy. While the mechanisms driving these immune cell count differences among populations are unclear, we speculate that females in industrialized populations may experience more dramatic vacillation in hormone production across the lifespan. Finally, we argue that differences in the magnitude and sometimes direction of sex biases in immune measures between the USA and the Tsimane show that current understanding of these processes is largely limited to the post-industrialized contexts where they have been predominantly studied. We therefore advocate that future research consider the impact of socio-ecological conditions and reproductive history on the physiological and behavioral processes that produce sexual dimorphism in immune function and disease risk.

## Materials and Methods

### The Tsimane

The Tsimane are subsistence-oriented horticulturalists inhabiting the Bolivian Amazonian River basin (census population ~ 17,000). Among the Tsimane, chronic exposure to diverse pathogens causes high infectious disease morbidity and mortality across all ages^67^, while incidence of allergies, atopy, autoimmune disease, obesity, and atherosclerosis is low^69^. As a result of elevated pathogen burden, Tsimane individuals exhibit high levels of immune activation compared to Western clinical standards^65,66^. Such high investment in immune function results in trade-offs with growth during development^81^ and high resting metabolic rate during adulthood^82^, reflecting the substantial energetic demands of coping with pathogenic threats. The Tsimane are also a “natural fertility” population, with limited access to effective contraception and breastfeeding alternatives^83^. As a result, Tsimane women have an average of 9 live births over the reproductive lifespan^84^ and exhibit nearly ubiquitous rates of on-demand breastfeeding following parturition, with a mean infant weaning age of 27 months^85^.

### Datasets

We used cross-sectional and longitudinal clinical and demographic data collected by the Tsimane Health and Life History Project (http://tsimane.anth.ucsb.edu/index.html)^32,65,66,86,87^ covering the period between 2004 and 2014. Approval by the Gran Consejo Tsimane and by institutional review boards at the University of California, Santa Barbara, the University of New Mexico, and the Universidad Mayor San Simon, Cochabamba Bolivia was obtained prior to data collection. Informed consent was provided by participants during a community-wide meeting open to all Tsimane residents and again at the individual level before each medical visit and interview. In the case of minors, parental consent was given before data were collected. Total leukocyte count was obtained via venous blood draws and determined with a QBC Autoread Plus dry hematology system (QBC Diagnostics). Relative fractions of neutrophils, eosinophils, lymphocytes, and monocytes were then measured manually by microscopy with a hemocytometer. Menarche was based on self-reported presence/absence of the first menstrual cycle. Pregnancy status was determined during medical visits based on the date of last menses, with urinary pregnancy tests administered by the physician when pregnancy was suspected. Pregnancies were cross-validated against subsequent annual demographic and census interviews, allowing detection of pregnancies that occurred between medical visits and pregnancies that went undetected during previous physician examinations^86^. Menopause was determined based on reported absence of a menstrual cycle over the past 6 months.

To obtain a representative sample from the United States, we used publicly available cross-sectional NHANES data collected by the Centers for Disease Control (https://www.cdc.gov/nchs/nhanes/index.htm) between 2003 and 2016. Total and differential leukocyte counts were measured using the Coulter method. Females who were under the age of 8 (for which reproductive data were redacted) or who specifically reported absence of menarche were binned as premenarchal. Females who were not currently pregnant, had not given birth within the preceding 12 months, and who reported a regular menstrual cycle either at time of exam or within the preceding two months were binned as regularly cycling. Out of 1,385 regularly cycling females with data on at least one immune marker, a subset (n = 504) had data on current use of hormonal birth control, with only 120 women reporting current use of hormonal birth control. Because data were missing for so many individuals, we did not include this variable in our statistical models. Females who self-reported being pregnant and/or had a positive urine test at the time of exam were categorized as currently pregnant. Women who were not currently pregnant but had given birth within the past 12 months were considered postpartum. Finally, females who reported absence of regular menstruation due to menopause were binned as postmenopausal.

### Sample Selection

Final THLHP and NHANES data sets were limited to males and females ages 2 to 84 with recorded white blood cell differential and body mass index (BMI). NLR was calculated by dividing neutrophil count by total lymphocyte count. We excluded 80 females from the NHANES dataset who reported reaching menopause before age 40 and 1 individual who reported regular menstruation after the age of 70 (effectively removing outliers associated with very early versus delayed menopause and/or errors in the data). We also removed extreme values for immune cell counts and NLR by limiting our sample to values which fell between population-pooled 1% and 99% percentiles. This exclusion criterion resulted in 3,956 data points (1.42%) being removed out of the initial pooled dataset consisting of 277,616 unique values. Given the ubiquity of chronic parasitic infections among the Tsimane, we did not exclude individuals with known infection or illness. In both populations, BMI varies considerably by age and sex (Figure S1), so we z-scored all BMI values using sex and age-specific mean values. No exclusions were based on medical diagnoses. Finally, we used the *matchit* package^88^ to create matched THLHP and NHANES samples stratified by age, sex, and female reproductive phase (specifying nearest neighbor matching on propensity score) (Figure S2, Supplementary Table 1).

### Statistical Analyses

All models were executed in R 4.3.2 (https://cran.r-project.org) using the *mgcv* package^89^. We employed generalized additive models (GAMs) to estimate the non-linear population-specific effects of age by sex and female reproductive phase (premenarchal, regularly cycling, pregnant, postpartum, postmenopausal) on total leukocyte, neutrophil, total lymphocyte, eosinophil, and monocyte count and neutrophil-to-lymphocyte ratio (NLR). All models also accounted for the fixed effects of z-scored BMI and the smoothed effects of parity, stratified by female reproductive phase (males and premenarchal females were all assigned parity values of zero). Due to repeat sampling of individuals within the THLHP dataset (Supplementary Table 2), all THLHP-specific models accounted for the group-level random effects of participant identification number.

## Supplementary Material

Supplementary Files

This is a list of supplementary files associated with this preprint. Click to download.

- womenshealthsupplement.docx

## Figures and Tables

**Table 1:** Description of immune markers used in this study.

Measure	Abbreviation	Description
Total white blood cells	WBC	The total number of circulating white blood cells (leukocytes).
Neutrophils	NEU	Most abundant type of white blood cell involved in myriad immune processes (e.g., antigen presentation, phagocytosis, priming of antigen-specific immune response). Implicated in autoimmune disease^90^.
Total lymphocytes	LYM	White blood cells characterized by the presence of the CD45 receptor and responsible for generating antigen-specific immune responses. Composed of T cells, B cells, and natural killer cells.
Eosinophils	EOS	Granulocytes that defend against macro-parasites and contribute to allergic responses.
Monocytes	MON	Phagocytic white blood cells that migrate to sites of infection and injury where they differentiate into macrophages.
Neutrophil-Lymphocyte Ratio	NLR	General marker of immune system homeostasis and the balance between innate and antigen-specific immunity. Values above 3 are generally considered to indicate inflammation^9^. Predictive of all-cause and cardiovascular mortality among US adults with rheumatoid arthritis^10^ and presence/severity of preeclampsia during pregnancy^91^, multiple sclerosis^11,12^, primary Sjogren's syndrome^13^, thyroid cancer^92^, and systemic lupus erythematosus^14^.

**Table 2:** Ages at which the 95% credible intervals for estimated values between males and females do not overlap, separated by population, measure, and female reproductive phase. Estimated values presented in this table are standardized by parity (set to 0 live births for premenarchal females and 3 live births for regularly cycling, pregnant, postpartum, and postmenopausal females) and z-scored BMI (set to 0).

Population	Measure	Reproductive Phase	Sex Bias	Age Range	Cumulative Years
Tsimane	LYM	Premenarchal	Male	2–3	2
Tsimane	MON	Premenarchal	Female	10–11	2
USA	LYM	Premenarchal	Female	2–4	3
USA	LYM	Premenarchal	Female	11–11	1
USA	EOS	Premenarchal	Male	2–9	8
USA	MON	Premenarchal	Male	3–9	7
Tsimane	WBC	Cycling	Male	14–24	11
Tsimane	NEU	Cycling	Male	14–22	9
Tsimane	LYM	Cycling	Male	14–26	13
Tsimane	EOS	Cycling	Male	14–18	5
Tsimane	NLR	Cycling	Female	14–14	1
USA	WBC	Cycling	Female	14–18	5
USA	WBC	Cycling	Female	43–48	6
USA	NEU	Cycling	Female	14–18	5
USA	NEU	Cycling	Female	40–50	11
USA	EOS	Cycling	Male	14–50	37
USA	MON	Cycling	Male	19–50	32
USA	NLR	Cycling	Female	43–50	8
Tsimane	WBC	Pregnant	Male	17–21	5
Tsimane	LYM	Pregnant	Male	17–31	15
Tsimane	EOS	Pregnant	Male	24–32	9
Tsimane	NLR	Pregnant	Female	18–42	25
USA	WBC	Pregnant	Female	17–40	24
USA	NEU	Pregnant	Female	17–41	25
USA	LYM	Pregnant	Male	31–41	11
USA	EOS	Pregnant	Male	17–44	28
USA	NLR	Pregnant	Female	20–42	23
Tsimane	MON	Postpartum	Female	35–47	13
USA	MON	Postpartum	Male	20–47	28
Tsimane	EOS	Postmenopausal	Female	58–59	2
Tsimane	NLR	Postmenopausal	Male	79–84	6
USA	NEU	Postmenopausal	Male	55–60	6
USA	LYM	Postmenopausal	Female	47–70	24
USA	EOS	Postmenopausal	Male	54–75	22
USA	MON	Postmenopausal	Male	40–83	44
USA	NLR	Postmenopausal	Male	50–83	34

## Data Availability

All NHANES data and R code used to conduct the statistical analyses for this paper are published on a public GitHub repository (https://github.com/carmenhove/sex_differences) and Zenodo (https://doi.org/10.5281/zenodo.19156930). Individual-level THLHP data are stored in the THLHP Data Repository and are available through restricted access for ethical reasons. THLHP’s highest priority is the safeguarding of human subjects and minimization of risk to study participants. The THLHP adheres to the “CARE Principles for Indigenous Data Governance” (Collective Benefit, Authority to Control, Responsibility, and Ethics), which assure that the Tsimane (i) have sovereignty over how data are shared, (ii) are the primary gatekeepers determining ethical use, (iii) are actively engaged in the data generation, and (iv) derive benefit from data generated and shared for use whenever possible. The THLHP is also committed to the “FAIR Guiding Principles for scientific data management and stewardship” (Findable, Accessible, Interoperable, Reusable). Requests for individual-level data should take the form of an application that details the exact uses of the data and the research questions to be addressed, procedures that will be used for data security and individual privacy, potential benefits to the study communities, and procedures for assessing and minimizing stigmatizing interpretations of the research results (see the following webpage for links to the data sharing policy and data request forms: https://tsimane.anth.ucsb.edu/data.html). Requests for individual-level data will require institutional IRB approval (even if exempt) and will be reviewed by an Advisory Council composed of Tsimane community leaders, community members, Bolivian scientists, and the THLHP leadership. The study authors and the THLHP leadership are committed to open science and are available to assist interested investigators in preparing data access requests.
